# A Missense Variant in the Bardet-Biedl Syndrome 2 Gene (*BBS2*) Leads to a Novel Syndromic Retinal Degeneration in the Shetland Sheepdog

**DOI:** 10.3390/genes12111771

**Published:** 2021-11-08

**Authors:** Rebekkah J. Hitti-Malin, Louise M. Burmeister, Frode Lingaas, Maria Kaukonen, Inka Pettinen, Hannes Lohi, David Sargan, Cathryn S. Mellersh

**Affiliations:** 1 Kennel Club Genetics Centre, Animal Health Trust, Lanwades Park, Newmarket, Suffolk CB8 7UU, UK; Rebekkah.Hitti-Malin@radboudumc.nl (R.J.H.-M.); cm935@cam.ac.uk (C.S.M.); 2 Department of Veterinary Medicine, University of Cambridge, Cambridge CB3 0ES, UK; drs20@cam.ac.uk; 3 Faculty of Veterinary Medicine and Biosciences, Department of Medical Genetics, Norwegian University of Life Sciences, P.O. Box 369 Sentrum, N-0102 Oslo, Norway; frode.lingaas@nmbu.no; 4 Department of Veterinary Biosciences, Department of Medical and Clinical Genetics, University of Helsinki and Folkhälsan Research Center, 00014 Helsinki, Finland; maria.kaukonen@helsinki.fi (M.K.); inka.pettinen@helsinki.fi (I.P.); hannes.lohi@helsinki.fi (H.L.)

**Keywords:** canine, PRA, retinal degeneration, BBS, *BBS2*, syndromic

## Abstract

Canine progressive retinal atrophy (PRA) describes a group of hereditary diseases characterized by photoreceptor cell death in the retina, leading to visual impairment. Despite the identification of multiple PRA-causing variants, extensive heterogeneity of PRA is observed across and within dog breeds, with many still genetically unsolved. This study sought to elucidate the causal variant for a distinct form of PRA in the Shetland sheepdog, using a whole-genome sequencing approach. Filtering variants from a single PRA-affected Shetland sheepdog genome compared to 176 genomes of other breeds identified a single nucleotide variant in exon 11 of the Bardet–Biedl syndrome-2 gene (*BBS2*) (c.1222G>C; p.Ala408Pro). Genotyping 1386 canids of 155 dog breeds, 15 cross breeds and 8 wolves indicated the c.1222G>C variant was only segregated within Shetland sheepdogs. Out of 505 Shetland sheepdogs, seven were homozygous for the variant. Clinical history and photographs for three homozygotes indicated the presence of a novel phenotype. In addition to PRA, additional clinical features in homozygous dogs support the discovery of a novel syndromic PRA in the breed. The development and utilization of a diagnostic DNA test aim to prevent the mutation from becoming more prevalent in the breed.

## 1. Introduction

Inherited retinal degenerations (IRDs) represent a group of hereditary diseases associated with reduced retinal function, leading to visual impairment and eventually blindness. In the domestic dog (*Canis familiaris*), progressive retinal atrophy (PRA) describes a group of heterogeneous IRDs characterized by depletion of rod and cone photoreceptor cells in the retina over time. The progression rate of PRA is variable, along with the etiology and age of onset, which can be broadly defined as early or late onset. Upon ophthalmoscopic evaluation, clinical signs of PRA include the attenuation of retinal blood vessels, widespread hyperreflectivity of the tapetum due to retinal thinning and atrophy of the optic disc, revealing the presence of bilateral retinal degeneration. In later stages, cataracts may develop secondary to the primary disease. PRA affects many dog breeds and is considered a welfare concern as vision loss is inevitable. Canine PRA is akin to human retinitis pigmentosa (RP) that affects 1 in 4000 humans worldwide [1], and shares phenotypic similarities.

Furthermore, many of the genes associated with canine IRDs have been implicated in human IRDs. This genetic overlap, accompanied by similarities between the human and canine eye morphology and size, means that the dog eye can be a useful model for human retinal research. Naturally occurring canine models exist for several IRDs, some of which have contributed to gene augmentation therapies in humans, such as the *Rpe65* model for Leber congenital amaurosis (LCA) [2], and the *Pde6a* [3] and *Pde6b* [4] models for autosomal recessive RP phenotypes. Currently, 271 genes have been implicated in a variety of human IRDs [5]. Human IRDs constitute several retinal disorders, including nonsyndromic diseases, such as RP, and syndromic diseases, including Bardet–Biedl syndrome (BBS), which involves a wide array of additional symptoms as well as retinal degeneration. Of the reported human IRD genes, 35 genes are also associated with canine IRDs. Nine genes are currently unique to canine IRDs but could be considered candidates for human IRDs. Twenty-nine genes have been implicated in canine PRAs, including two genes previously associated with human BBS [6,7]. Despite identifying many human and canine IRD genes, many individuals of both species still lack a molecular diagnosis.

While the effectiveness of gene therapy has proven successful as a treatment for some retinal degenerations in canine models, PRA is currently incurable. Therefore, the use of DNA testing is crucial in controlling the disease in canine populations, offering dog breeders a tool to determine their dog’s genotype for a specific PRA-associated variant. This knowledge enables dog breeders to avoid breeding clinically affected puppies, even before clinical signs of the disease become visible, as well as identify heterozygotes of a recessive condition.

In the last decade, whole-genome sequencing (WGS) has become increasingly used in disease variant identification to compare affected (case) and non-affected (control) individuals. With WGS analysis, various filtering steps must be performed to strategically decide which variants to consider for involvement with the disease. The use of canine genome banks facilitates WGS analysis, enabling further variant filtration whereby, a single genome can be used as a case in one study and a control genome for other studies. Although the majority of canine PRA-associated variants found are located within coding regions of the genome, disease-causing variants in non-coding areas have been identified [8,9,10]. WGS, as opposed to a targeted sequencing approach (including whole exome sequencing; WES) enables the entire genome to be interrogated and provides a comprehensive dataset.

The British Veterinary Association/Kennel Club/International Sheep Dog Society (BVA/KC/ISDS) Eye Scheme is a canine clinical eye screening scheme, which lists known hereditary ocular diseases and the breeds affected with these conditions, based upon the consistent opinion of veterinary ophthalmologists in the UK. Dogs intended to be used for breeding are advised to undergo annual eye examinations to determine if they are clinically affected or unaffected by the disease(s) listed for their breed. Before 2018, the Shetland sheepdog (SS) was listed on this scheme as being affected by collie eye anomaly (CEA) and PRA. Although the SS is no longer formally listed as being affected by PRA under the scheme, the disease nevertheless remained a concern for the English SS Breed Club in the UK when this genetic study was initiated because a small number of cases had been identified through routine ophthalmoscopic screening. One form of PRA has previously been described in the SS, caused by an exonic 4-base pair (bp) deletion in the cyclic nucleotide gated channel α 1 gene (*CNGA1*) [11]. However, genetic heterogeneity had been observed in the study cohort. Several SS unrelated to the *CNGA1*-affected proband were genetically clear of the deletion, suggesting that at least one additional form of PRA was segregating in the breed. This assumption was further supported when additional SSs in the UK population were clinically diagnosed with PRA, yet were also genetically clear of the *CNGA1* deletion. Knowledge of non-*CNGA1* forms of PRA in the breed, with an unknown number of carriers in worldwide populations, warranted further study of PRA to identify genetically unsolved cases. One PRA-affected SS was submitted to the Kennel Club Genetics Center at the Animal Health Trust (AHT) for WGS analysis. This study aimed to identify the genetic cause of a novel form of PRA in the SS, to both improve understanding of the etiology of this disease and enable the development of a DNA test for breeders to use as a tool to reduce the frequency of, and eventually eradicate, a novel form of PRA in the breed.

## 2. Materials and Methods

### 2.1. Sample Collection

PRA in the SS was diagnosed based on the ophthalmoscopic evaluation by a board-certified veterinary ophthalmologist or a qualified BVA/KC/ISDS eye scheme panelist (or the European equivalent if outside the UK). Buccal mucosal cheek swabs were used to collect DNA from PRA cases and controls (approved by the AHT ethics committee; ref no. 24-2018E). Samples from a litter of three SS dogs, comprising one bitch (proband SS1) and two dogs (probands SS2 and SS3), were submitted to the Kennel Club Genetics Center at the Animal Health Trust due to a PRA diagnosis in the bitch. Control dogs were selected as those aged 8 years and over that had been examined through the BVA/KC/ISDS eye scheme and determined to be clinically clear of PRA. DNA was extracted from buccal mucosal swabs using the QIAamp DNA Blood Mini Kit (Qiagen, Manchester, UK) according to the manufacturer’s instructions. DNA concentration and purity were determined using the NanoDrop 1000 spectrophotometer (Thermo Fisher Scientific, Loughborough, UK) and/or the Qubit Fluorometer with the Qubit dsDNA broad range (BR) assay kit (Invitrogen, Loughborough, UK).

Additional SS samples were collected and genotyped for the candidate variant by The University of Helsinki, Finland (*n* = 394) and The Norwegian University of Life Sciences, Norway (*n*= 8). The University of Pennsylvania, USA generously shared DNA from a family of nine SSs to also be genotyped for the candidate variant. Phenotype information available for SS dogs concluded that: one PRA case, two SSs with suspected PRA, and two SSs affected with other forms of retinopathy were present within The University of Helsinki cohort; all eight SSs in The Norwegian University of Life Sciences cohort were PRA cases, and one PRA case was present within The University of Pennsylvania DNA samples.

### 2.2. Whole-Genome Sequencing (WGS)

Before WGS, 27 variants that were previously published and associated with canine retinal diseases, including the *CNGA1* variant identified in the SS breed, were excluded as mutations responsible for this form of PRA in the SS, using a multiplex PCR followed by sequencing or genotyping method (Table S1). Genomic DNA from a SS PRA case was normalized to 25 ng/μL, and 1000 ng was sent for sequencing, outsourced to Edinburgh Genomics, UK. Illumina sequencing of a TruSeq Nano library on a HiSeq X sequencing platform (Illumina) generated a dataset of approximately 30x coverage of the dog genome. Reads were aligned to the canine reference genome (CanFam3.1) using BWA-MEM [12], variant calls were made using the Genome Analysis Tool Kit (GATK) v3.6 (HaplotypeCaller) and base quality score recalibration, insertion/deletion (indel) realignment and duplicate removal were performed [13]. SNP and indel discovery were performed using standard hard filtering parameters or variant quality score recalibration according to GATK best practice recommendations [14,15]. Genomic Variant Call Format (gVCF) files from 186 canine WGSs (in the AHT genome bank) were merged by CombineGVCFs (GATK) into a multi-sample Variant Call Format (VCF) file. The cross-genome analysis was conducted using Variant Effect Predictor (VEP) by Ensembl (Dog release 89) [16] on a merged VCF file. WGS reads were visualized in Integrative Genomics Viewer (IGV) software [17]. Chromosomal positions of eight additional previously published PRA-associated variants (Table S1) that were not screened prior to performing WGS were visualized in the SS WGS data to determine genotypes.

### 2.3. Variant Filtering

An in-house effect score was assigned to each variant depending on its predicted impact on the protein-coding sequence, and whether it was predicted to be deleterious (Table S2). Variants were filtered for those with a high effect score (effect score 4 and 5), including those resulting in premature start/stop codons, splice site variants, nonsense and missense variants, frameshift variants and in-frame insertions and deletions. High effect variants were initially filtered for those segregating as an autosomal recessive disease. An X-linked dominant mode of inheritance was also considered by filtering high impact variants on the X chromosome. Further variant filtering was conducted using the Dog Biomedical Variant Database Consortium (DBVDC) [18] as a canine genome bank to filter out common polymorphisms that were frequent across multiple dog breeds. Variants were also prioritized based on the gene implicated and its candidacy. A list of keywords related to PRA was compiled and cross-referenced through the OMIM database to extract genes present in the WGS analysis that were associated directly or indirectly with any words in the PRA keyword list (see File S1). This highlighted genes with previous evidence of an association with ocular phenotypes and prioritized high impact variants based on gene function. To assess the impact of the candidate variant amino acid substitution on gene protein function, in silico bioinformatic tools Mutation Analyzer [19], PolyPhen-2 (Polymorphism Phenotyping v2) [20,21] and SIFT (Sorting Intolerant From Tolerant) [22] were used to predict the effect of the identified variant computationally.

### 2.4. Extracting All Variants in BBS Genes

The syndromic phenotype of proband SS1 led to the exploration of potential genetic correlates of BBS. To highlight additional variants in other genes associated with human BBS, all variants regardless of their assigned effect score were extracted from the genomic VCF file to a text file. The following ‘grep’ Unix command was used to extract all variants that were located in 21 known BBS genes, including gene synonyms in the search: grep -wE“(Chr|BBS1|BBS2|ARL6|BBS4|BBS5|MKKS|BBS7|BBS8|TTC8|BBS9|BBS10|TRIM32|BBS12|MKS1|CEP290|WDPCP|SDCCA8|LZTFL1|BBIP1|IFT27|IFT172|C8orf37|BBIP10)” input_file.txt > output_file.txt.

### 2.5. Variant Validation and Genotyping

The candidate single nucleotide variant (SNV) in *BBS2* was confirmed using Sanger sequencing. PCR products to be used for Sanger sequencing were purified on a MultiScreen PCRμ96 filter plate (Merck Millipore, Watford, UK) and sequenced using the Sanger method using Bigdye v3.1 chemistry (Life Technologies Ltd., Loughborough, UK) and the following conditions: 96 °C for 30 s; 44 cycles at 92 °C for 4 s; 55 °C for 4 s; and 60 °C for 1 min 50 s. To remove excess reagents, isopropanol precipitation of sequencing reaction products was performed. Precipitated DNA was resuspended in 10 µL Hi-Di Formamide (Applied Biosystems, Loughborough, UK) and sequencing products were separated on an Applied Biosystems (ABI) 3130xl Genetic Analyzer. Sequencing reads for each sample were assembled using the Staden package [23]. The *CEP290* SNV was genotyped using PCR followed by Sanger sequencing, as described above.

An allelic discrimination probe-based assay was designed to effectively distinguish between individuals that were homozygous for the *BBS2* SNV (C/C), heterozygous (G/C) or homozygous for the wild-type/common allele (G/G). Allelic discrimination assays were performed using the following qPCR probes and primers: qPCR probe 1 5′-HEX-ACACCATCATCCGAGCAGTACTGA- IBFQ -3′; qPCR probe 2 5′-FAM-ACACCATCATCCGACCAGTACTGA- IBFQ -3′; primer 1 5′-CCATCTTGGACTGGTTCTTG; primer 2 5′-CTGGAAAGGTTGTGAATGCT. Probes were PrimeTime ZEN double-quenched qPCR probes containing a 5′ fluorophore, 3′ Iowa Black^®^ FQ (IBFQ) quencher and proprietary internal ZEN™ quencher. A 5′ HEX™ fluorophore was used to determine the reference allele and a FAM™ fluorophore to label the alternate allele (purchased from Integrated DNA Technologies (IDT); Leuven, Belgium) as PrimeTime assays. Real-time PCR was used to amplify template DNA using Luna universal qPCR Master Mix (New England Biolabs (NEB) Ltd., Herts, UK) on a StepOnePlus™ real-time PCR system (ABI). The following cycling parameters were used: 25° for 30 s, 95° for 3 min, 35 cycles of 95° for 3 s, 60° for 10 s and a final post-PCR holding step at 25° for 30 s. Results were analyzed using ABI StepOne Software v2.3 (ABI). The University of Helsinki, Finland, genotyped a cohort of SS dogs (*n* = 394, including one PRA-affected SS; samples collected under the permission of the Animal Experimental Board of Regional State Administrative Agency of Southern Finland, ESAVI/7482/04.10.07/2015, ESAVI/25696/2020) using the allelic discrimination assay described above. The Norwegian University of Life Sciences, Norway, also sequenced SS PRA cases submitted to their research for the identified variant using the Sanger method and primers and conditions described above. The University of Pennsylvania, USA also provided DNA from SS to contribute towards variant validation.

## 3. Results

### 3.1. Phenotypes

Of the three related SSs in the original litter, a PRA diagnosis was confirmed in probands SS1 and SS2 by a BVA/KC/ISDS panelist and/or veterinary ophthalmologist. Proband SS1 was 8.7 years old at the time that the PRA diagnosis was confirmed. The same proband had been examined under the BVA/KC/ISDS eye scheme 16 months earlier (aged 7.4 years) by the same veterinary ophthalmologist and was clinically unaffected at that time. PRA was diagnosed based on bilateral retinal degeneration findings due to tapetal hyperreflectivity with retinal vascular attenuation and a pale optic disc. Menace and pupillary light responses were present but reduced, and no cataracts were present. Night blindness had been apparent for four weeks before the confirmed diagnosis and a noticeable decline in daylight vision followed thereafter. A maze test was performed where proband SS1 collided with obstacles under scotopic conditions but avoided obstacles under photopic conditions. An extended profile electroretinogram showed depressed rod and cone response. Ophthalmoscopic follow-up examination at 11.3 years reported no PRA progression, but cataract formation at the equatorial region. Mature cataracts were present bilaterally by the age of 12 years. Veterinary records showed that proband SS2 was also diagnosed with retinal abnormalities consistent with PRA by a board-certified veterinary ophthalmologist. This included tapetal hyperreflectivity and bilateral retinal blood vessel attenuation at the age of six years. Although proband SS3 was not formally examined through the BVA/KC/ISDS eye scheme or by a board-certified veterinary ophthalmologist, a PRA diagnosis was suspected. The owners of proband SS3 reported that the dog started to suffer visual impairment around the age of 9–10 years, initially presenting with reduced vision in low light conditions, which progressed until his death at the age of 15 years. The dam of the litter was examined under the BVA/KC/ISDS scheme at 11.4 years and was clinically unaffected. There is no evidence that a veterinary ophthalmologist examined the sire’s eyes.

Veterinary history regarding weight was only available from the age of 11 years for proband SS1. Weight was recorded from the age of 11 years until euthanasia at the age of 13.9 years, ranging from 5.1 kg–6.6 kg (normal range for the breed is 6.8 kg–11.3 kg), with the owner reporting that the dog had been on a low-fat diet since her earlier years. Proband SS2 was deemed obese, weighing 19.5 kg at the age of 5.5 years. The owner reported that proband SS2 had a severely increased appetite and described the dog as ‘ravenous’ when offered food. A low-fat diet eventually reduced the dog’s weight to 14 kg at the age of 6.4 years, but this was not maintained, resulting in subsequent weight gain. A dirlotapide-based drug was used from the age of 5.9 years in an attempt to reduce body weight. No information on the weight of proband SS3 was available.

Limited information was available on renal biochemistry for proband SS1. However, nephroliths were present at 13.3 years of age and eventually the owner elected for euthanasia at 13.9 years due to renal failure. For proband SS2, urea and creatine levels were within normal limits at 5.5 years of age: 7.9 moles per liter (mol/L) (normal range 2.5–9.6 mol/L) and 85 mol/L (normal range 44–159 mol/L), respectively. When assessed at 8 years of age, these levels had increased (urea 26.7 mol/L; creatine 160 mol/L). Two months later, urea and creatine levels had increased dramatically to 35.6 mol/L and 258 mol/L, respectively, resulting in renal failure. The veterinarian prescribed a renal diet feed and Fortekor^®^ to aid in the management of chronic renal failure. By the age of 9.4 years, kidney disease had progressed with elevated urea and creatine levels (urea 46.4 mol/L; creatine 444 mol/L) and the owner elected for euthanasia. No information on the renal health of proband SS3 was available.

**Figure 1:**
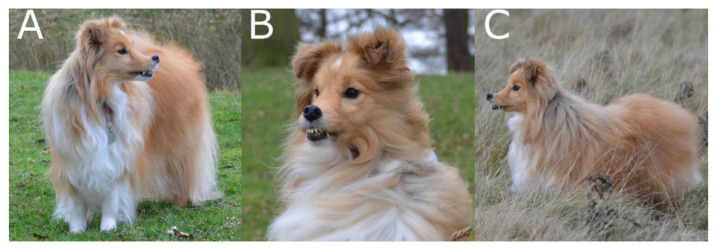
Photographs of PRA-affected proband SS1 in the initial litter show the uncharacteristic ‘wavy’ coat, upturned nose and dental defects (**A**–**C**).

Proband SS1 showed uncharacteristic features for the breed, including an upturned nose, an abnormal coat of a wavy texture and dental abnormalities (Figure 1). These features became increasingly noticeable as the dog developed, becoming more apparent around the age of six months. Veterinary history also noted dental remarks where multiple teeth were extracted at the age of 11.9 years as lower incisors were loose. No further information on the physical appearance of proband SS2 was available, although the dog was reported to have undergone dental treatment of three teeth extractions due to resorptive lesions. Similarly, no further information was available on the physical appearance of proband SS3. Photographs of both sire and dam show they did not exhibit the unusual nose shape or coat texture observed in the affected offspring.

### 3.2. WGS Variant Filtering

The exclusion of 35 previously published canine retinal mutations, including the *CNGA1* SS variant, indicated the presence of a novel PRA-associated mutation. At the time of the study, 185 canine genomes, sequenced as part of other studies in our laboratory were available for WGS analyses. The merged VCF file contained 186 genomes (1 case versus 185 controls) with a total of 27,611,812 variants amongst all genomes. Of these 185 genomes, 176 were of dogs recruited for non-PRA studies, and these were considered PRA controls for the purposes of this study. Filtering for variants present in the 176 PRA control genomes and with a high effect score (4 or 5) retained 136,383 high impact variants. As the litter consisted of one PRA-affected female, one PRA-affected male and one male suspected to be affected with PRA, an X-linked dominant mode of inheritance was considered. However, as the sire was unaffected and the dam was clinically confirmed as unaffected, an X-linked dominant PRA was unlikely. Nevertheless, IGV software was used to visualize sequencing reads from proband SS1 over the entire *RPGR* gene, which has previously been implicated in canine X-linked PRA [24]. No variants of interest were present in *RPGR*. In addition, no high impact variants segregated appropriately for an X-linked mode of inheritance (dominant or recessive) or were present in six genes associated with X-linked RP in humans: *RP2* [25,26], *OFD1* [27,28,29], *TIMM8A* [30], *CACNA1F* [31,32] and *NYX* [33,34,35]. Thus, an X-linked model was excluded, and an autosomal recessive mode of inheritance was considered most likely. Filtering based on an autosomal recessive model, i.e., retaining high effect variants homozygous in proband SS1 and either heterozygous or homozygous for the alternate allele in all 176 non-breed matched controls, resulted in 151 variants.

Exclusion of variants located on unknown chromosomes further narrowed the variant list down to 134 variants. These 134 variants were checked across canine genomes in the DBVDC, which comprised of 648 genomes at the time of the study (32 belonging to the AHT), including three SS genomes. After filtering out common SNVs, 12 variants remained that were all homozygous in the SS case and were absent from all other genomes in the DBVDC (Table S3). Assessing the genes harboring these 12 variants in the OMIM database against a list of keywords considered to be associated with PRA phenotypes resulted in one deleterious variant in a gene associated in human IRDs (*BBS2*).

In parallel with screening the DBVDC, all genes in which the 151 variants were located were assessed for their candidacy: 74 annotated genes (80 variants) and 40 Ensembl canine stable identifiers (ENSCAF IDs; 71 variants). Of the 40 ENSCAF IDs, human homologues could be determined for ten genes (28 variants). Fifty-seven variants located within ENSCAF IDs of which no human orthologous region was present and/or those on unknown chromosomes were excluded. Therefore, a total of 84 gene names (accounting for 94 variants) were investigated in the OMIM database against a list of keywords considered to be associated with PRA phenotypes. Thirty-eight variants were predicted to be tolerated by SIFT and 37 variants were homozygous in multiple non-SS or non-Collie breed control genomes in the DBVDC and were therefore excluded. The remaining 19 variants were manually visualized in IGV following de novo local realignment using HaplotypeCaller [36], which confirmed that 14 variants were called correctly by variant annotation tools (Table S4). All 14 variants were investigated further.

### 3.3. Variant Mining

**Figure 2:**
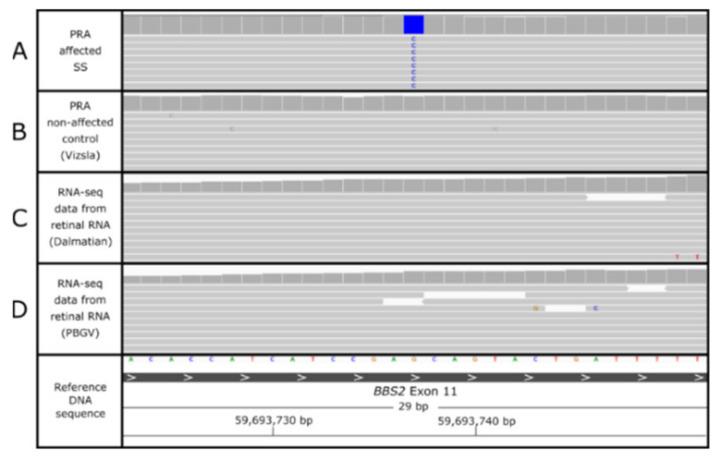
IGV display of WGS reads aligned across the *BBS2* c.1222G>C SNV region. Grey horizontal bars show paired-end sequencing reads aligned to the canine reference genome across the *BBS2* exon 11 location. Above these sequencing reads is a histogram representing sequencing coverage across the region. Track (**A**) shows the WGS reads from the PRA-affected SS (proband SS1). The blue block shows a homozygous SNV change from the reference nucleotide ‘G’ to a ‘C’ at position chr2:59693737. Track (**B**) shows WGS data from a PRA non-affected control genome (Hungarian Vizsla dog) that is homozygous for the reference ‘G’ allele. *BBS2* is expressed in retinal tissue as demonstrated by RNA-seq alignment from two control dogs: one Dalmatian dog (track (**C**)) and one Petit Basset Griffon Vendéen (PBGV) dog (track (**D**)), both of which are also homozygous for the reference allele at this position.

The 14 variants identified in the variant filtering process were genotyped across a cohort of 43 SS controls, which were aged 8 years or older and diagnosed as clinically unaffected by PRA. PCR amplicons could only be generated for 12 variants, among which 11 were homozygous in at least one of the SS controls and therefore were excluded as causal PRA variants. Two PCR amplicons failed sequencing, probably due to difficulty in amplification due to repetitive sequence and/or a GC-rich sequence. These were variants in the genes *C7* and *IRS2*. RNA sequencing (RNA-seq) data generated for an unrelated study from a PRA non-affected Petit Basset Griffon Vendéen canine retina [37] showed *C7* and *IRS2* were considered to be highly expressed in the canine retina. Although genotyping-by-sequencing of the *C7* and *IRS2* variants failed, the variants were identified in additional breeds in the DBVDC genomes. The variant in *C7* was present in two SS control dogs in homozygous and heterozygous states and was also heterozygous in WGS from a Lancashire heeler dog. No sequencing data were available for the *IRS2* variant in multiple genomes and two Dachshund dogs were heterozygous for the insertion. Table S4 summarizes the 14 variants and determines whether they could be excluded as candidate variants or not. One candidate variant is a homozygous missense SNV in the Bardet–Biedl syndrome 2 (*BBS2*) gene (Figure 2), situated on canine chromosome 2 (CANFA2). Although the variants in *GGN*, *C7* and *IRS2* could not be entirely excluded in control SSs using PCR methods, the candidacy of *BBS2* warranted prioritization of the *BBS2* SNV for further exploration.

### 3.4. BBS2 c.1222G>C Missense Variant

**Figure 3:**
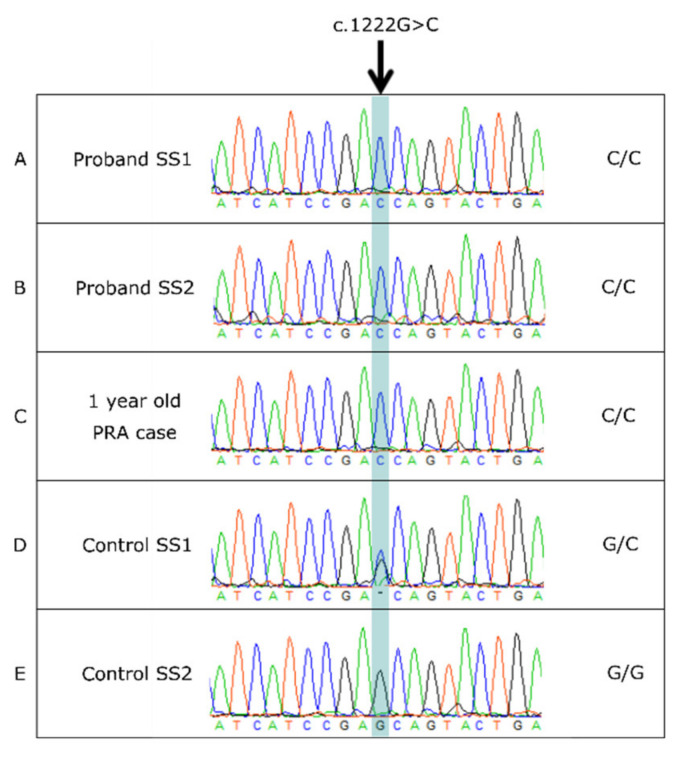
Sanger sequencing of the *BBS2* c.1222G>C variant confirmed its presence in proband SS1 (track (**A**)). Additional dogs were also homozygous for the c.1222G>C variant (tracks (**B**,**C**)). SSs heterozygous for the variant and homozygous for the wild-type allele (G/G) are shown in tracks (**D**) and (**E**), respectively.

Sanger sequencing confirmed the c.1222G>C missense SNV in exon 11 of *BBS2* (Figure 3). This non-synonymous variant results in an amino acid change from alanine to proline at amino acid position 408, p.(Ala408Pro). Mutation Analyzer and PolyPhen-2 predicted the *BBS2* c.1222G>C, p.Ala408Pro amino acid change to be rarely substituted and probably damaging to the protein, respectively (PolyPhen-2 score 0.993). SIFT predicted the change to be deleterious with a score of 0.03.

### 3.5. BBS2 c.1222G>C Population Screening

To better understand the frequency and predicted causality of c.1222G>C, the variant was searched in: (a) different SS cohorts; (b) in SS PRA cases clear of the *CNGA1* mutation and non-affected cases; (c) in different breeds of dogs. In total, c.1222G>C was genotyped across 1386 canids of 155 dog breeds, 15 cross breeds and 8 wolves (Table S5). This included 505 SS, of which 91 were submitted directly to the AHT research database. Frequencies of the c.1222G>C variant in tested SS cohorts are detailed in Table 1.

Taken together with the AHT cohort, a total of 14 SS were confirmed PRA cases, with four individuals homozygous for the c.1222G>C variant (probands SS2 and SS3 from the AHT original litter; two SS from the Norwegian cohort), and ten were homozygous for the reference/common allele (G/G). Pedigree information from the two homozygous dogs from the Norwegian cohort showed that they were non-related within five generations to the original WGS case. No photographs or information were available regarding any additional phenotypic information. All genotypes of SSs tested for the c.1222G>C variant are summarized in Table 2. This constitutes dogs that were either confirmed PRA cases, controls known to be PRA non-affected or dogs with an unknown PRA diagnosis.

Of the 858 dogs of non-SS breeds genotyped for the variant, 136 were Border Collie dogs, including six PRA cases. These were, and remain, genetically unsolved, with the c.1222G>C variant only identified in SSs in the present study. As genotyped in this study, allele frequencies for the c.1222G>C variant are summarized in Table 3.

### 3.6. Characterization of the BBS2-PRA Phenotype

Genotyping of two SSs, aged 1 year and 7.6 years old, with the same distinctive characteristics as the initial case (upturned nose, unusual coat and dental defects) revealed that both are homozygous for the c.1222G>C variant. The 7.6-year-old underwent an ophthalmoscopic examination by a board-certified ophthalmologist (who was also a BVA/KC/ISDS panelist), however no clinical signs of PRA were observed. Pedigree information for these two additional homozygotes showed that both dogs share common ancestry with probands SS1, SS2 and SS3 in the initial SS litter (Figure S1). The pedigree also highlighted third-generation relatives of the proband, which were excluded in determining allele frequencies so that the data were more representative of the general SS population.

Phenotype information for the seven homozygous SSs is summarized in Table 4, demonstrating shared phenotypic features in addition to the PRA phenotype to characterize the BBS2-PRA phenotype.

### 3.7. Additional BBS Gene Variants

Multiple BBS-associated variants have been described in human cases, including biallelic and monoallelic variants, with heterozygous variants in up to three genes (reviewed by [38]). To assess whether an oligogenic phenotype was manifesting in the affected SS, any variants (regardless of their predicted effect on the coding sequence) residing in one of the 21 genes known to be implicated in human BBS were extracted for further investigation. A total of 510,752 variants were present in the VCF output file for the genome of proband SS1, of which 174 variants met these criteria. Of the 174 variants, 3 variants were situated in the 3′ untranslated region (of BBS10), 17 were intergenic variants and 152 intronic variants. The only exonic variants were the c.1222G>C SNV in *BBS2*, present only in the SS, and a missense variant in *CEP290* (c.3011G>A; p.(Ser1003Leu)). The *CEP290* SNV was homozygous in three additional breeds in the AHT genome bank (one Border Collie crossbreed, one Bulldog and one Smooth Collie) and homozygous and/or heterozygous in 19 dogs of 14 breeds in the DBVDC. The *CEP290* SNV was also present in dbSNP (rs851859358) with a minor allele frequency of 0.021, yet no phenotype information was available. The variant was tolerated by SIFT with a score of 0.26. Five SSs homozygous for the *BBS2* c.1222G>C variant, one heterozygous SS and one SS homozygous for the wild-type allele were genotyped for the *CEP290* SNV; all were homozygous for the alternate ‘A’ allele at this position.

## 4. Discussion

WGS analysis of a single PRA-affected SS, compared to 176 non-breed matched control genomes, revealed a SNV in the *BBS2* gene located on CANFA2. The *BBS2* c.1222G>C SNV segregated appropriately for an autosomal recessive disorder in the SS breed, with a predicted severe impact on the protein. Genotyping of additional dogs demonstrated that the SNV was not observed in any other breeds, indicating the variant was supposed to be private to the SS breed. Further to proband SS1 sent for WGS, additional c.1222G>C homozygous SS were identified, including two full siblings to the proband. Where available, clinical information and photographs confirmed a PRA diagnosis and/or additional clinical features. The involvement of *BBS2* in human BBS patients sharing similar clinical features presents *BBS2* as a compelling candidate gene for a novel syndromic form of PRA in the SS.

Human BBS is classically an autosomal recessive multisystem ciliopathy. There is a wide array of clinical manifestations whereby a BBS diagnosis is based on the presence of at least four primary features, or three primary and two (or more) secondary features [39]. In human patients, primary features of the condition include retinal degeneration, polydactyly, obesity, genital abnormalities, renal abnormalities and learning difficulties. A diverse spectrum of secondary features manifest, including speech and developmental delays, diabetes, dental defects, congenital heart disease, brachydactyly and polydactyly, ataxia and craniofacial abnormalities, including an abnormally high arched palate and depressed nasal bridge [39]. While human BBS phenotypes are characterized by loss of function mutations in 24 genes [40], all involved in the primary cilia functioning, many encoding genes involved in BBS specifically regulate the machinery of the multi-subunit complex, termed the BBSome. The BBSome consists of eight highly conserved proteins, BBS1-9 and BBS18/BBIP10, which function in primary cilium biogenesis [41,42]. BBS proteins control the formation of the primary cilia by facilitating cargo movement between plasma and ciliary membranes. This process occurs across various cell types where primary cilia are present, including the retina and kidneys. The BBS2, BBS7 and BBS9 proteins form the BBSome core complex, which is essential in BBSome maturation [43]. Structurally, BBS2, BBS7 and BBS9 are homologous proteins that share distinct protein folding and domain organization [44]. Within the BBS2-7-9 subcomplex, BBS2 is located between BBS7 and BBS9 with the α-helical domain of BBS2 in contact with BBS9 [45].

The canine *BBS2* gene encodes a 715 amino acid protein with three domains (Figure S2). The N-terminal is predicted to be positioned from amino acid residues 22–120; a mid-terminal from 159–266 and a C-terminal from 270–709 (Pfam; [46]) with a putative coiled-coil domain between 317–344 amino acid residues [47]. The c.1222G>C; p.Ala408Pro SNV identified in the SS PRA case resides within the predicted C-terminal domain of *BBS2*. *BBS2* is conserved across species showing 90% sequence similarity to the human orthologue. Additionally, the ‘GCA’ codon implicated is well conserved across 37 mammals, including humans. Variants in *BBS2* are associated with BBS [48,49,50] and nonsyndromic RP [51] in humans, and have been implicated in retinal phenotypes in mice [52] and zebrafish [53]. To date, 94 pathogenic or likely pathogenic variants and 158 variants of unknown significance (VUS) have been reported in *BBS2* in humans [54]. The canine p.Ala408 is at the orthologous region in the human BBS2 gene p.Ala414, and although no pathogenic/likely pathogenic variants have been reported at this position, three pathogenic/likely pathogenic variants have been reported in exon 11, including a nonsense SNV at c.1237C>T (p.Arg413Ter) [55]. The *BBS2* p.Ala408Pro in the present study is predicted to be positioned within the C-terminal of BBS2. Variants reported within the human BBS2 C-terminal include a *BBS2* mutation causing an amino acid substitution of arginine to proline at residue 632 (p.Arg632Pro) reported in BBS patients [55,56,57]. Binding experiments of the p.Arg632Pro substitution revealed that the mutation is located in the α-helical domain of BBS2 [45]. Ludlam et al. [45] demonstrated that BBS2 interacts with BBS7, forming a tight dimer that associates with the rest of the BBSome hexameric subcomplex (comprised of BBS1, 4, 5, 8, 9, 18). Therefore, the p.Arg632Pro substitution likely causes BBS because of an inability of the BBS2/7 dimer to bind to BBS9 and the rest of the BBSome [45]. Thus, considering the results of aforementioned studies establishing the presence of the α-helical domain of BBS2 within the C-terminal [45,57], it may be hypothesized that this candidate SNV results in a loss of function of BBS2 in the canine BBS2-7-9 complex, disrupting BBSome assembly, which may impact ciliary transport machinery in the complex.

Retinal photoreceptor primary cilia and outer segments are essential for phototransduction and maturation of the RPE, suggesting that the c.1222G>C variant impairs retinal function through dysfunction of the phototransduction cascade. We described SSs diagnosed with bilateral retinal degeneration based on a PRA-like appearance, and additional c.1222G>C homozygotes were also clinically affected with PRA. Exceptions to this include proband SS3 in the initial litter, which was reported blind with PRA-like symptoms by the owner but was not confirmed by a veterinary ophthalmologist. In addition, the two SSs later identified as c.1222G>C homozygotes were unrelated within three-generations to probands SS1, SS2 and SS3, aged 1 year and 7.6 years. Where a PRA diagnosis was made, the age of onset varied. Therefore, both the 1-year-old and 7.6-year-old c.1222G>C homozygotes will most likely develop this form of PRA within their lifetimes. Routine ophthalmoscopic follow up of c.1222G>C homozygotes will determine disease age of onset and progression. In addition to PRA, dogs harboring two c.1222G>C alleles showed a distinctive upturned nose, which is uncharacteristic of the breed, possibly due to a depressed nasal bridge as described in human BBS [39]. Other subtle craniofacial secondary features of human BBS include undeveloped cheekbones (malar hypoplasia), deep-set eyes with short or narrow palpebral fissures, a wide forehead and jaw malformations [39,58,59], for which clinical data for these features were unavailable for the SSs in this study. Notably, at least some of the c.1222G>C homozygous dogs were on a low-fat diet for much of their adult life, with one classed as severely obese. Furthermore, one case showed signs of increased motivation for food, described by the owner as having a “voracious” appetite. Due to limited clinical information, there is insufficient evidence to support the hypothesis of an association between the *BBS2* c.1222G>C variant and an increased appetite or obesity. However, it is plausible that these dogs have dysregulation of satiety hormones, such as leptin due to dysfunction of BBS2 in the BBSome, resulting in leptin resistance and therefore an increased food intake leading to weight gain, as demonstrated in BBS knockout mouse models [60,61]. Further study may determine whether weight is a contributing factor or whether the c.1222G>C homozygotes were overweight due to overfeeding. Primary cilia, on the surface of the nephron’s epithelial cells and collecting duct in the renal cortex of the kidney, plays a role in epithelial homeostasis and repair. Furthermore, human BBS patients with variants in the genes encoding the BBS2-7-9 complex have a high frequency of kidney anomalies, suggesting this complex is more critical for the kidney’s function and development than other BBSome components [40]. Information on kidney health was not available for all probands in the study. However, kidney malfunctions were observed, including one SS being on a kidney support diet and medication for renal disease. Potential renal defects may be explained by ciliogenesis in the kidneys and the specific role of the BBS2-7-9 complex. All additional features mentioned above are shared amongst human BBS phenotypes [38,62,63,64,65]. The exception to this is the abnormal hair phenotype exhibited in SSs, which could be due to primary cilia’s involvement in hair follicle development and homeostasis. Relatable findings have been demonstrated in the Oak Ridge Polycystic Kidney (ORPK) mouse as a model for cystic kidney disease and cilia dysfunction, whereby ORPK mice display a scruffy fur phenotype in addition to renal defects, retinal degeneration due to a loss of photoreceptor outer segments, polydactyly and other similarities to severe BBS (dental crowding, abnormal palatal morphology, skeletal defects) (reviewed by [66]). Therefore, further study of the BBS2 canine phenotype is required to understand the array of clinical manifestations described in the SS PRA cases.

Previous research demonstrates similarities between BBS-null mice and canine PRA phenotypes, specifically in the Hungarian Puli dog where a nonsense SNV in *BBS4* causes a novel syndromic PRA [7], with phenotypes comparable to Bbs4-null mice [50]. In addition to PRA, *BBS4*-affected Hungarian Puli dogs displayed spermatozoa flagella defects, albeit less severely affected than that observed in Bbs4-null mice, as well as obesity. This was the first BBS-like phenotype reported in dogs. Before this, the only BBS gene implicated in canine PRA was the *TTC8* gene, also known as *BBS8*, in Golden Retriever dogs [6]. Although dogs in the initial *TTC8* study by Downs et al. [6] were not reported to have other clinical and morphological features in addition to PRA, it has since been shown that the 1-bp deletion in *TTC8* leads to a syndromic PRA phenotype, with dogs exhibiting signs of obesity, renal anomalies, sperm defects and olfactory defects [67]. For the SS, no information regarding spermatozoa flagella potency in male dogs was available to conclude if fertility defects are present. However, for proband SS5, the owner reported that the bitch did not have a season until past the breed’s typical age, around the age of 5 years. Further follow-up of canine *BBS2* models is required to understand the spectrum and complexity of additional features, ideally throughout the dog’s lifetime with the ability to monitor retinal and renal changes as well as weight tracking and fertility programs. As with the *BBS4* and *TTC8* canine phenotypes, it is plausible that the *BBS2* c.1222G>C SNV causes a syndromic PRA in the SS. Typically, when studying canine PRA, a diagnosis is limited to ophthalmoscopic evaluation. Distinguishing between a canine nonsyndromic and syndromic condition requires recognition of multiple clinical signs, where additional features can be challenging to quantify or assess. Changes in social dominance, olfactory defects and learning or developmental delay are some of the behavioral and neurological features displayed in BBS human patients and mouse models, which are challenging to quantify in dogs. Further to this, obesity is a common problem in dogs, whereby 39–65% of dogs are classified as overweight and between 9–20% are obese [68,69]. Therefore, its association with a genetic disorder can easily be overlooked. Physical facets, including craniofacial or dental features, are more pronounced; however, renal deficits would need to be monitored by a veterinary surgeon to characterize. In future studies of canine PRA, BBS genes’ involvement should be considered, particularly when a diagnosis is based on ophthalmoscopy alone and information regarding physical or additional characteristics is limited.

As oligogenic inheritance has been observed in human BBS [57], this prospect was explored in the current study. The only notable exonic variant found to segregate in addition to the *BBS2* SNV was a SNV in *CEP290*. CEP290 interacts with numerous ciliary proteins and has been implicated in human BBS type-14 [70] in addition to a nonsyndromic IRD, LCA [71]. The *CEP290* SNV was present in SS of all three BBS2 genotypes (with five SS homozygous for the *BBS2* SNV also homozygous for the *CEP290* SNV) in addition to multiple dogs of other breeds, suggesting that this is probably an ancestral polymorphism. However, further genotyping of SSs is required to confirm this. It appears that the BBS-phenotype in the SS is a single-gene recessive disorder caused by the exonic *BBS2* c.1222G>C SNV, however, to exclude the involvement of intronic variants as potential oligogenic contributors, such as triallelic or modifier mutations, targeted sequencing of all BBS genes across numerous c.1222G>C homozygotes and non-affected SS controls, or functional cDNA or RNA analysis on tissue from an affected dog would be required. Furthermore, this analysis did not exclude the presence of intronic variants that may impact splicing, including potential deep-intronic variants in other genes, due to the compelling variant in *BBS2*.

The allele frequency of 0.021, when excluding third-generation relatives to the proband from available pedigree information, indicates this is a rare variant and at a low frequency in the current SS population. Despite this, sample collection showed the variant is still present in the current UK population and SS populations elsewhere, and the availability of a DNA test (termed BBS2-PRA) will raise awareness of this form of PRA. Utilization of such a diagnostic test within the SS breeding community will reduce the possibility of future generations becoming affected with this form of PRA, with the potential to eliminate this form of PRA from the gene pool possibly within the next few generations. The use of a DNA test would provide genetic information on the status of this PRA-associated mutation before clinical signs present, and therefore provide a tool for breeding choices before dogs reach breeding age. Moreover, the absence of *BBS2* c.1222G>C in additional PRA cases in the screening cohort suggests at least one other form of PRA in the breed remains unsolved. This provides an opportunity to explore PRA in the SS breed further.

## Figures and Tables

**Table 1:** Frequencies of the *BBS2* c.1222G>C variant in various SS cohorts. ‘G’ represents the wild-type/common allele, ‘C’ represents the alternate allele.

Cohort	c.1222G>C C/C	c.1222G>C G/C	c.1222G>C G/G	Total SS Genotyped
AHT	5	11	75	91
The University of Helsinki	0	6	388	394
The Norwegian University of Life Sciences	2	0	6	8
The University of Pennsylvania	0	0	9	9
DBVDC	0	0	3	3
Total SS genotyped				505

**Table 2:** Genotype distribution of the *BBS2* c.1222G>C SNV in SS confirmed PRA cases and non-affected controls or SS with an unknown PRA diagnosis.

	Confirmed PRA Cases			PRA Non-Affected			Unknown PRA Diagnosis		
c.1222G>C genotype	C/C	G/C	G/G	C/C	G/C	G/G	C/C	G/C	G/G
Number of individuals	4	0	10	2 ^†^	11	29	1	6	442

^†^ These PRA non-affected dogs that are homozygous for the *BBS2* c.1222G>C SNV are aged 1 year and 7.6 years old. Due to the late-onset nature of this form of PRA, these dogs will probably develop PRA in their lifetimes. Both dogs presented with additional characteristics of the *BBS2* phenotype (see probands SS4 and SS5 in Table 4).

**Table 3:** Allele frequency of *BBS2* c.1222G>C SNV in multiple breeds of dog. ‘G’ represents the wild-type/common allele, ‘C’ represents the alternate allele.

Breed	c.1222G>C Genotype			Allele Frequency of Causal Allele
	C/C	G/C	G/G	
SS	7	17	481	0.031
SS *	4	13	481	0.021
Crossbreeds	0	0	15	0
Wolves	0	0	8	0
154 other breeds	0	0	858	0

* Excluding third-generation relatives to the proband.

**Table 4:** Summary of the SS homozygous for the *BBS2* c.1222G>C variant and their phenotypes.

Proband	Ocular Phenotype	Age of Diagnosis	Age at Submission	Additional Phenotypic Features
SS1	PRA confirmed	8.7 years	9 years	- Upturned nose - Abnormal coat - Dental abnormalities. - Kidney issues - On a low-fat diet but weight managed
SS2	PRA confirmed	6 years	9 years	- No photographic evidence available for physical characteristics - Weight issues (clinically obese) and severely increased appetite - Kidney health issues
SS3	PRA unconfirmed but blind	9–10 years	9 years	- No photographic evidence available for physical characteristics - No clinical history regarding renal or weight status available
SS4	No ophthalmoscopic examination	N/A	1 year	- Upturned nose - Abnormal coat - Dental abnormalities - No clinical history regarding renal or weight status available
SS5	PRA unaffected	7.6 years	7.6 years	- Upturned nose - Abnormal coat - Dental abnormalities - Severely increased appetite - Possible fertility disorder - No clinical history regarding renal status available
SS6	PRA confirmed	Unknown	Unknown	- No photographic evidence available for physical characteristics - No clinical history regarding renal or weight status available
SS7	PRA confirmed	Unknown	Unknown	- No photographic evidence available for physical characteristics - No clinical history regarding renal or weight status available

## Data Availability

WGS data for proband 1 is deposited in the European Nucleotide Archive: https://www.ebi.ac.uk/ena/browser/view/PRJEB44362.

## Supplementary Materials

The following are available online at https://www.mdpi.com/article/10.3390/genes12111771/s1. Table S1: Variants associated with canine inherited retinal degenerations (IRDs) and the methods used to exclude each variant as causal in proband SS1 before whole-genome sequencing. Table S2: In-house effect scores assigned to sequence ontology terms used to prioritize variant filtration in whole-genome sequencing analysis. Table S3: A list of twelve variants homozygous in the affected dog that remained after filtering out non-common variants using the DBVDC. An asterisks (*) highlights those variants overlapping with the 15 variants in the final filtering list following candidate gene analysis in Table S4. Table S4: Fourteen variants identified through WGS filtering to exclude in a SS cohort. Table S5: The number of individuals from each dog breed screened for the BBS2 c.1222G>C and each sample origin. Figure S1: Pedigree drawing of proband SS1 shows there is shared ancestry between five *BBS2* c.1222G>C homozygotes. Male dogs are shown as a square symbol and females as a circle symbol. Individuals colored pink are related within three generations to the proband SS1. Figure S2: A schematic of the canine BBS2 protein. The N-terminal (BBS2_N) is positioned between amino acid residues 22-120. The middle region domain (BBS2_M) is located between amino acids 159-266. The C-terminal (BBS2_C) resides between amino acids 270-709 with a putative coiled-coil domain (CC) from 317-344 amino acids. The BBS2 candidate SNV affects amino acid position 408, as highlighted by a red asterisk. File S1: List of keywords associated with progressive retinal atrophy (PRA).
