# Supplementary material for: A Missense Variant in the Bardet-Biedl Syndrome 2 Gene (BBS2) Leads to a Novel Syndromic Retinal Degeneration in the Shetland Sheepdog
Source: Genes (Basel). 2021 Nov 8;12(11):1771. doi: 10.3390/genes12111771 (PMC8624581; doi:10.3390/genes12111771)
Supplement: Supplementary file 1 [file genes-12-01771-s001.zip › genes-1425830-SP/references for Table S1.pdf]

1. Mellersh CS, Boursnell ME, Pettitt L, Ryder EJ, Holmes NG, Grafham D, et al. Canine RPGRIP1 mutation establishes cone-rod dystrophy in miniature longhaired dachshunds as a homologue of human Leber congenital amaurosis. Genomics. 2006;88(3):293-301.
2. Forman OP, Hitti RJ, Boursnell M, Miyadera K, Sargan D, Mellersh C. Canine genome assembly correction facilitates identification of a MAP9 deletion as a potential age of onset modifier for RPGRIP1-associated canine retinal degeneration. 2016;27(5-6):237-45.
3. Kropatsch R, Akkad DA, Frank M, Rosenhagen C, Altmüller J, Nürnberg P, et al. A large deletion in RPGR causes XLPRA in Weimaraner dogs. Canine Genetics and Epidemiology. 2016;3(1).
4. Wiik AC, Wade C, Biagi T, Ropstad EO, Bjerkas E, Lindblad-Toh K, et al. A deletion in nephronophthisis 4 (NPHP4) is associated with recessive cone-rod dystrophy in standard wire-haired dachshund. Genome Res. 2008;18(9):1415-21.
5. Goldstein O, Mezey JG, Schweitzer PA, Boyko AR, Gao C, Bustamante CD, et al. IQCB1 and PDE6B mutations cause similar early onset retinal degenerations in two closely related terrier dog breeds. Invest Ophthalmol Vis Sci. 2013;54(10):7005-19.
6. Dekomien G, Runte M, Godde R, Epplen JT. Generalized progressive retinal atrophy of Sloughi dogs is due to an 8-bp insertion in exon 21 of the PDE6B gene. Cytogenetics and cell genetics. 2000;90(3-4):261-7.
7. Clements PJ, Gregory CY, Peterson-Jones SM, Sargan DR, Bhattacharya SS. Confirmation of the rod cGMP phosphodiesterase β subunit (PDE β) nonsense mutation in affected rcd-1 Irish setters in the UK and development of a diagnostic test. Current eye research. 1993;12(9):861-6.
8. Suber ML, Pittler SJ, Qin N, Wright GC, Holcombe V, Lee RH, et al. Irish setter dogs affected with rod/cone dysplasia contain a nonsense mutation in the rod cGMP phosphodiesterase β-subunit gene. Proc Natl Acad Sci U S A. 1993;90(9):3968-72.
9. Kukekova AV, Goldstein O, Johnson JL, Richardson MA, Pearce-Kelling SE, Swaroop A, et al. Canine RD3 mutation establishes rod-cone dysplasia type 2 (rcd2) as ortholog of human and murine rd3. Mamm Genome. 2009;20(2):109-23.
10. Petersen-Jones SM, Entz DD, Sargan DR. cGMP phosphodiesterase-α mutation causes progressive retinal atrophy in the Cardigan Welsh corgi dog. Invest Ophthalmol Vis Sci. 1999;40(8):1637-44.
11. Downs LM, Bell JS, Freeman J, Hartley C, Hayward LJ, Mellersh CS. Late-onset progressive retinal atrophy in the Gordon and Irish Setter breeds is associated with a frameshift mutation in C2orf71. Anim Genet. 2013;44(2):169-77.
12. Goldstein O, Kukekova AV, Aguirre GD, Acland GM. Exonic SINE insertion in STK38L causes canine early retinal degeneration (erd). Genomics. 2010;96(6):362-8.
13. Dekomien G, Vollrath C, Petrasch-Parwez E, Boeve MH, Akkad DA, Gerding WM, et al. Progressive retinal atrophy in Schapendoes dogs: mutation of the newly identified CCDC66 gene. Neurogenetics. 2010;11(2):163-74.
14. Ahonen SJ, Arumilli M, Lohi H. A CNGB1 frameshift mutation in Papillon and Phalene dogs with progressive retinal atrophy. PLoS One. 2013;8(8):e72122.
15. Goldstein O, Zangerl B, Pearce-Kelling S, Sidjanin DJ, Kijas JW, Felix J, et al. Linkage disequilibrium mapping in domestic dog breeds narrows the progressive rod-cone degeneration interval and identifies ancestral disease-transmitting chromosome. Genomics. 2006;88(5):541-50.
16. Zangerl B, Goldstein O, Philp AR, Lindauer SJ, Pearce-Kelling SE, Mullins RF, et al. Identical mutation in a novel retinal gene causes progressive rod-cone degeneration in dogs and retinitis pigmentosa in humans. Genomics. 2006;88(5):551-63.
17. Kijas JW, Cideciyan AV, Aleman TS, Pianta MJ, Pearce-Kelling SE, Miller BJ, et al. Naturally occurring rhodopsin mutation in the dog causes retinal dysfunction and degeneration mimicking human dominant retinitis pigmentosa. Proc Natl Acad Sci U S A. 2002;99(9):6328-33.
18. Kijas JW, Miller BJ, Pearce-Kelling SE, Aguirre GD, Acland GM. Canine models of ocular disease: outcross breedings define a dominant disorder present in the English mastiff and bull mastiff dog breeds. J Hered. 2003;94(1):27-30.
19. Downs LM, Wallin-Hakansson B, Boursnell M, Marklund S, Hedhammar A, Truve K, et al. A frameshift mutation in golden retriever dogs with progressive retinal atrophy endorses SLC4A3 as a candidate gene for human retinal degenerations. PLoS One. 2011;6(6):e21452.
20. Goldstein O, Jordan JA, Aguirre GD, Acland GM. A non-stop S-antigen gene mutation is associated with late onset hereditary retinal degeneration in dogs. Mol Vis. 2013;19:1871-84.

21. Murgiano L, Becker D, Torjman D, Niggel JK, Milano A, Cullen C, et al. Complex Structural PPT1 Variant Associated with Non-syndromic Canine Retinal Degeneration. G3 (Bethesda). 2018.
22. Zhang Q, Acland GM, Parshall CJ, Haskell J, Ray K, Aguirre GD. Characterization of canine photoreceptor phosducin cDNA and identification of a sequence variant in dogs with photoreceptor dysplasia. Gene. 1998;215(2):231-9.
23. Everson R, Pettitt L, Forman OP, Dower-Tylee O, McLaughlin B, Ahonen S, et al. An intronic LINE-1 insertion in MERTK is strongly associated with retinopathy in Swedish Vallhund dogs. PLoS One. 2017;12(8):e0183021.
24. Yeh CY, Goldstein O, Kukekova AV, Holley D, Knollinger AM, Huson HJ, et al. Genomic deletion of CNGB3 is identical by descent in multiple canine breeds and causes achromatopsia. BMC Genet. 2013;14:27.
25. Tanaka N, Dutrow EV, Miyadera K, Delemotte L, MacDermaid CM, Reinstein SL, et al. Canine CNGA3 Gene Mutations Provide Novel Insights into Human Achromatopsia-Associated Channelopathies and Treatment. PLoS One. 2015;10(9):e0138943.
26. Veske A, Nilsson SE, Narfstrom K, Gal A. Retinal dystrophy of Swedish briard/briard-beagle dogs is due to a 4-bp deletion in RPE65. Genomics. 1999;57(1):57-
27. Guziewicz KE, Zangerl B, Lindauer SJ, Mullins RF, Sandmeyer LS, Grahn BH, et al. Bestrophin gene mutations cause canine multifocal retinopathy: a novel animal model for best disease. Invest Ophthalmol Vis Sci. 2007;48(5):1959-67.
28. Zangerl B, Wickstrom K, Slavik J, Lindauer SJ, Ahonen S, Schelling C, et al. Assessment of canine BEST1 variations identifies new mutations and establishes an independent bestrophinopathy model (cmr3). Mol Vis. 2010;16:2791-804.
