# Supplementary material for: A Missense Variant in the Bardet-Biedl Syndrome 2 Gene (BBS2) Leads to a Novel Syndromic Retinal Degeneration in the Shetland Sheepdog
Source: Genes (Basel). 2021 Nov 8;12(11):1771. doi: 10.3390/genes12111771 (PMC8624581; doi:10.3390/genes12111771)
Supplement: Supplementary file 1 [file genes-12-01771-s001.zip › genes-1425830-SP/BBS2_PRA_supplementary_revisions_submit_19102021.pdf]

genes

SUPPLEMENTARY FILES

Table S2: In-house effect scores assigned to sequence ontology terms used to prioritise variant filtration in whole genome sequencing analysis.

Sequence ontology term	Effect Score
5′-UTR premature start codon gain variant	5
Chromosome number variation	5
Disruptive in-frame deletion	5
Disruptive in-frame insertion	5
Exon loss	5
Exon loss variant	5
Frameshift variant	5
In-frame deletion	5
In-frame insertion	5
Initiator codon variant	5
Initiator codon variant and non-canonical start codon	5
Missense variant	5
Protein altering variant	5
Rare amino acid variant	5
Splice acceptor variant	5
Splice donor variant	5
Start lost	5
Stop gained	5
Stop lost	5
Transcript ablation	5
3′-UTR truncation	4
5 ‘-UTR truncation	4
Non-coding exon variant	4
Regulatory region ablation	4
Splice region variant	4
TFBS ablation	4
Coding sequence variant	3
Conserved intergenic variant	3
Conserved intron variant	3

Sequence ontology term	Effect Score
3 prime UTR variant	2
5 prime UTR variant	2
Exon variant	2
Feature elongation	2
Feature truncation	2
Gene variant	2
Intron variant	2
Mature microRNA (miRNA) variant	2
miRNA	2
Nonsense mediated decay (NMD) transcript variant	2
Non-coding transcript exon variant	2
Non-coding transcript variant	2
Regulatory region amplification	2
Regulatory region variant	2
Start retained	2
Stop retained variant	2
TFBS variant	2
TFBS amplification	2
Transcript amplification	2
Transcript variant	2
Upstream gene variant	2
Coding sequence variant	1
Downstream gene variant	1
Intergenic region	1
Intergenic variant	1
Intragenic variant	1
Synonymous variant	1
Transcript	1

Table S3: A list of twelve variants homozygous in the affected dog that remained after filtering out non-common variants using the DBVDC. An asterisks (*) highlights those variants overlapping with the 15 variants in the final filtering list following candidate gene analysis in Table S4.

Variant	CanFam3.1 Chromosomal Position	Gene/Ensembl Stable Identifier	Variant	In-house effect score	SIFT score
V1*	2:59693737	*BBS2*	Missense SNV	5	Deleterious (0.03)
V2*	X:43769675	*BMP15*	Missense SNV	5	Deleterious (0.01)
V3	11:12969395	ENSCAFG00000012222 (human orthologue *EEF1A1*)	15-bp deletion	5	None
V4*	30:24115675	ENSCAFG00000016650 (human orthologue *PSMA1*)	Missense SNV	5	Deleterious (0)
V5	10:17263700	ENSCAFG00000028547 (no human orthologue)	3-bp deletion	5	None
V6	18:40776257	ENSCAFG00000029824 (no human orthologue)	Missense SNV	5	Deleterious (0.04)
V7	15:13802173	ENSCAFG00000031689 (no human orthologue)	Missense SNV	5	None
V8	38:23457534	ENSCAFG00000031841 (no human orthologue)	3-bp insertion	5	None
V9	17:4745695	ENSCAFG00000038172 (no human orthologue)	2-bp insertion	4	None
V10	4:5245679	*IRF2BP2*	Missense SNV	5	Tolerated low confidence (0.18)
V11*	X:1762952	*MXRA5*	Missense SNV	5	Deleterious (0.04)
V12	17:40080783	*TRABD2A*	Missense SNV	5	Deleterious_ low confidence (0.05)

Table S4: Fourteen variants identified through WGS filtering to exclude in a SS cohort. Filtering variants from WGS analysis identified 14 variants to follow up in additional SS controls. One of these variants is located in a gene associated with a retinal phenotype in humans, marked by an asterisk (*). Variants were checked for in genomes in the Dog Biomedical Variant Database Consortium (DBVDC) genome bank in addition to sequencing in 43 SS controls aged 8 years or older, and were excluded as variants of interest if homozygous in at least one control SS. Variants with low confidence in sequencing reads due to low coverage or those where control dogs were only heterozygous or homozygous for the alternate allele were not excluded as potential candidate variants.

Variant	CanFam3.1 Chromosomal Position	Gene/Ensembl Stable Identifier	Variant	Excluded by screening DBVDC	Excluded by screening 43 control SS
V1	1: 110147994	*CD3EAP*	Missense SNV	Not excluded	Excluded
V2	1: 116038553	ENSCAFG00000028805 (no human orthologue)	Missense SNV	Not excluded	Excluded
V3	1: 119547008	*ZNF507*	1-bp deletion	Not excluded	Excluded
V4	2: 62484625	*CHD9*	Missense SNV	Not excluded	Excluded
V5	15: 52265347	*FGG*	Splice site insertion of transposable element	Not excluded	Excluded
V6	16: 36561913	*DLC1*	Missense SNV	Not excluded	Excluded
V7	16: 44477295	*F11*	Splice site SNV	Not excluded	Excluded
V8	30: 24115675	ENSCAFG00000016650 (human orthologue *PSMA1*)	Missense SNV	Not excluded	Excluded
V9	36: 10135919	*SLC38A11*	Missense SNV	Not excluded	Excluded
V10	X: 1762952	*MXRA5*	Missense SNV	Not excluded	Excluded
V11	X: 43769675	*BMP15*	Missense SNV	Not excluded	Excluded
V12	4: 68451901	*C7*	Nonsense SNV	Not excluded	Not excluded
V13	22: 58226397	*IRS2*	3-bp insertion	Not Excluded	Not excluded
V14	2: 59693737	*BBS2**	Missense SNV	Not excluded	Not excluded

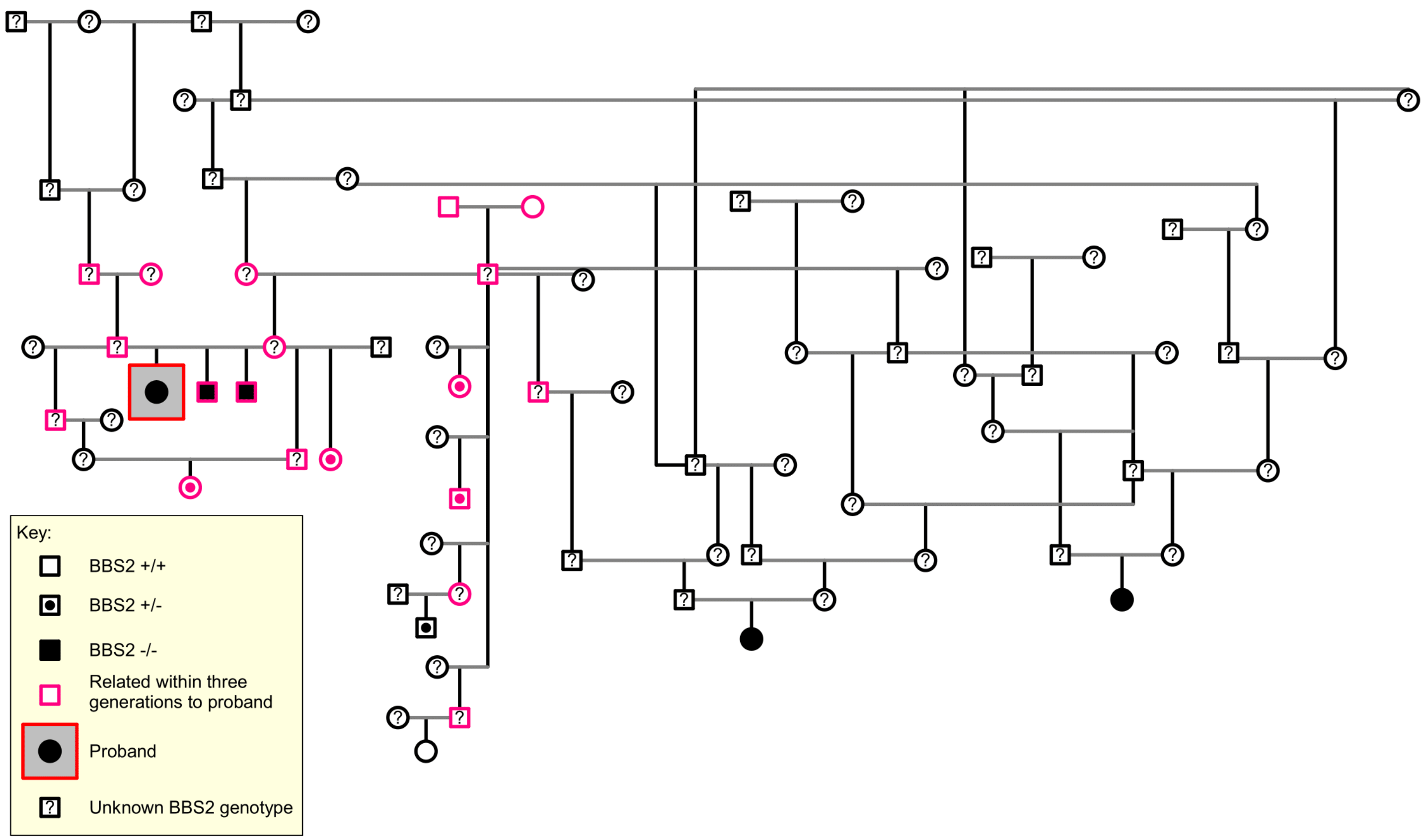

Figure S1: Pedigree drawing of proband SS1shows there is shared ancestry between five *BBS2* c.1222G>C homozygotes. Male dogs are shown as a square symbol and females as a circle symbol. Individuals coloured pink are related within three generations to the proband SS1.

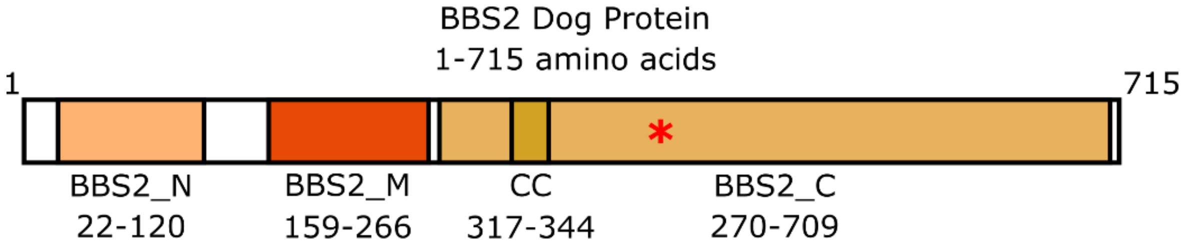

Figure S2: A schematic of the canine BBS2 protein. The N-terminal (BBS2_N) is positioned between amino acid residues 22-120. The middle region domain (BBS2_M) is located between amino acids 159-266. The C-terminal (BBS2_C) resides between amino acids 270-709 with a putative coiled-coil domain (CC) from 317-344 amino acids. The BBS2 candidate SNV affects amino acid position 408, as highlighted by a red asterisk.

List of keywords associated with progressive retinal atrophy (PRA).Retina, progressive retinal atrophy, retinitis pigmentosa, rod-cone dysplasia/rod cone dysplasia, cone-rod dysplasia/cone rod dysplasia, cone degeneration, retinal degeneration, rod dysplasia, photoreceptor, retinal dystrophy, retinal pigment epithelium, nyctalopia, bone spicules, fundus, blindness, Leber congenital amaurosis, Bardet-Biedl syndrome/Bardet Biedl syndrome, chorioretinal atrophy, chorioretinal degeneration, cone dystrophy, cone-rod dystrophy/cone rod dystrophy, rod-cone dystrophy/rod cone dystrophy, congenital stationary night blindness, macular degeneration, ocular-retinal developmental disease/ocular retinal developmental disease, optic atrophy, Usher syndrome, retinopathy, cilium, ciliopathy.
